# Relationship between Affective Symptoms and Malnutrition Severity in Severe Anorexia Nervosa

**DOI:** 10.1371/journal.pone.0049380

**Published:** 2012-11-21

**Authors:** Lama Mattar, Caroline Huas, EVHAN group, Nathalie Godart

**Affiliations:** 1 INSERM U669, Maison de Solenn, Paris, France; 2 Université Paris-Sud, Paris, France; 3 Université Paris Descartes, Paris, France; 4 Psychiatry Unit, Institut Mutualiste Montsouris 42, Paris, France; 5 Faculté de médecine Paris Descartes, Département Universitaire de Médecine Générale, Tours, France; University of Granada, Spain

## Abstract

**Background:**

Very few studies have investigated the relationship between malnutrition and psychological symptoms in Anorexia Nervosa (AN). They have used only body weight or body mass index (BMI) for the nutritional assessment and did not always report on medication, or if they did, it was not included in the analysis of results, and they did not include confounding factors such as duration of illness, AN subtype or age. The present study investigates this relationship using indicators other than BMI/weight, among which body composition and biological markers, also considering potential confounders related to depression and anxiety.

**Methods:**

155 AN patients, (DSM-IV) were included consecutively upon admission to inpatient treatment. Depression, anxiety, obsessive behaviours and social functioning were measured using various scales. Nutritional status was measured using BMI, severity of weight loss, body composition, and albumin and prealbumin levels.

**Results:**

No correlation was found between BMI at inclusion, fat-free mass index, fat mass index, and severity of weight loss and any of the psychometric scores. Age and medication are the only factors that affect the psychological scores. None of the psychological scores were explained by the nutritional indicators with the exception of albumin levels which was negatively linked to the LSAS fear score (p = 0.024; beta = −0.225). Only the use of antidepressants explained the variability in BDI scores (p = 0.029; beta = 0.228) and anxiolytic use explained the variability in HADs depression scores (p = 0.037; beta = 0.216).

**Conclusion:**

The present study is a pioneer investigation of various nutritional markers in relation to psychological symptoms in severely malnourished AN patients. The clinical hypothesis that malnutrition partly causes depression and anxiety symptoms in AN in acute phase is not confirmed, and future studies are needed to back up our results.

## Introduction

The severely malnourished and disturbed biochemical status of patients with Anorexia Nervosa (AN) [1] is a fundamental clinical and somatic aspect of the disorder. Clinical consensus agrees that psychological disturbances in AN patients, such as depression and anxiety symptoms, are partly complications of malnutrition [2]. Several hypotheses and mechanisms have been proposed to explain this impact; studies have shown implications of the serotonergic system in mood and depression symptoms; starved AN patients might be having low tryptophan intake, the precursor of serotonin, which is affecting their mood [3], [4]. Another hypothesis is the effect of low leptin levels in AN due to low adiposity [5], shown to have functional role in depression [6] anxiety and cognitive behaviour [7], [8]. Another approach is related to vitamins and minerals deficiencies and their replenishment. In fact, almost all vitamins have key roles in the brain functions and the nervous system. In the same time, vitamins deficiencies are very common and chronic in AN patients [9]. Other various theories have arisen concerning macronutrients intake, specifically carbohydrates and low carbohydrates diets affecting the mood and creating depression-like symptoms [10]. AN patients, tend to have very low carbohydrates diets and low fat diets, which might affect negatively their mood on the long term.

Despite this implication of malnutrition in the appearance of anxiety and depressive symptoms, [11] evidence-based data on this relationship in AN is still very scarce [12]. We recently reviewed all the studies that investigated this relationship in AN. Some simply observed an improvement in psychological condition during nutrition rehabilitation, while the others reported inconsistent findings with no correlation between malnutrition (weight/BMI) and psychological symptoms. Three limitations were found across most of the studies reviewed. Firstly, they used only body weight or body mass index (BMI) for the nutritional assessment [4]–[7]. Secondly, they did not always report on medication, or if they did, it was not included in the analysis of results. Lastly, they did not include confounding factors such as duration of illness, AN subtype or age. In fact the duration of the illness itself can lead to depressive symptoms, as in any chronic disease [13].

Nutritional assessment cannot be based solely on BMI or body weight [14]. The limitations of these methods of nutritional assessment have been outlined in our recent review. Although BMI is a widely accepted screening tool for obesity, its specificity and sensitivity in undernutrition are unknown [15]. In cases of severe malnutrition, body weight alone, like many other useful screening tools, is not sufficiently sensitive to determine nutritional status [9], [10]. Moreover, in children and adolescents, BMI should be used with caution, as it is relative to age; for instance a BMI of 17.5 would be on the 3rd percentile for a 19-year-old but on the 50th percentile for an 11-year old [16]. To our knowledge, no previous studies have investigated malnutrition using other indicators than BMI or weight, such as body composition components (fat mass and fat-free mass) and biological markers (albumin and prealbumin) and their relationship with the psychological status of AN patients, also considering factors related to depression and anxiety.

Several debatable questions arise following the above introduction: 1) Would body composition components such as fat mass and fat free mass or biological markers tell us more about the relationship between the nutritional status of AN patients and depression and anxiety symptoms? 2) Are psychological symptoms directly related to nutritional status markers? Therefore, we hypothesis that 1) Depression and anxiety symptoms present in AN patient at admission to inpatient treatment are related to the severity of malnutrition, to the intensity of weight loss before admission and to the duration of illness. 2) Body composition and biological markers describe better the nutritional status compared to BMI and might be linked to the psychological symptoms. Thus the aim of the present study is to investigate, among severe AN subjects admitted to inpatient treatment units, the link between nutritional status evaluated by 3 different parameters (BMI, body composition and biological markers) and the severity of depressive, anxiety, OCD and social phobia symptoms, adjusting for confounding factors such as duration of illness, AN subtype and medication.

## Methods

### Ethics Statement

This study was part of a larger study named EVHAN (evaluation of hospitalisation for AN, also named in French EVALHOSPITAM, Eudract number: 2007-A01110-53, registered in Clinical trials). This study protocol was approved by the Ile-de-France III Ethics Committee and the CNIL (Commission nationale de l’informatique et des libertés). Written informed consent was obtained from each patient before inclusion in accordance with the declaration of Helsinki.

### Subjects

One hundred and fifty-five consecutive female AN patients were included in this study between April 2009 and May 2011. The patients were recruited from the inpatient treatment facilities of 11 centres in France (CHU- Bordeaux, Cochin – Maison des Adolescents, Institut Mutualiste Montsouris, MGEN – La Verrière, CHU-Nantes, CHU-Rouen, Robert Debre Hospital, Sainte-Anne Hospital, Saint-Etienne Hospital, Villejuif – Paul Brousse). Current AN diagnosis was based on the DSM-IV criteria obtained by the Eating Disorder Examination (EDE) [17] and the CIDI 3.0 with the following BMI criteria: BMI <10^th^ percentile up to 17 years of age, and BMI<17.5 for 17 years of age and above [18].

At inclusion, four patients did not have a BMI<17.5. However 2 of them went from a BMI above the 97^th^ percentile, to a BMI on the 10^th^ percentile relative to their age in the year preceding hospitalization. The remaining 2 had a BMI<17.5 in the previous three months but were initially admitted to medicine wards, and had gained weight just before their transfer to the psychiatry unit and inclusion in the study. Three patients did not meet DSM_IV AN criterion D (amenorrhea, i.e. the absence of at least three consecutive menstrual cycles), but they reported irregularity in the cycles. In fact, amenorrhea is no longer a required criterion for AN diagnosis (DSM V) [19].

The overall assessment investigated different aspects concerning patient psychiatric/psychological and somatic status at admission to inpatient treatment. All the assessments were performed in the hospitals in the first 2 weeks after hospitalization.

### Assessments

#### 1 Psychological evaluation

The Beck Depression Inventory (BDI), a self-rating scale of 21 items assesses cognitive and motivational symptoms of depression at the time of evaluation [20], [21].

The Hospital Anxiety and Depression scale (HADs) is a self-report scale rating 14 items, which assesses the most frequent anxiety and depression symptoms. It also evaluates their severity, taking somatic aspects that might affect the rating into account [22], [23].

The Liebowitz Social Anxiety Scale (LSAS) is a clinical interview divided into 2 sets of questions concerning current fear and avoidance in social interaction and performance-oriented situations [24].

The Maudsley Obsessive Compulsive Inventory (MOCI) is a self-report questionnaire designed to assess obsessive-compulsive behavior using 30 items in true/false format classified in 4 sub-scales (Checking compulsions, Washing/cleaning compulsions, Slowness, Doubting) [25], [26].

#### 2 Nutritional assessment

Anthropometry: Body weight was measured with standard balance beam scales (SECA, Germany) to the nearest 0.1 kg in underwear. The subject stood squarely on the scales not touching anything. Height was measured with a stadiometer (SECA, Germany) to the nearest 0.1 cm. with the subject standing with heels together, arms to the sides, legs straight, shoulders relaxed and head in the horizontal plane (“look straight ahead”).

- The BMI in Kg/m^2^ was calculated as the weight in kilograms divided by the height in meters, squared.
- Severity of weight loss: estimated as the difference between the maximum BMI before illness and BMI at inclusion.

Body composition by bioelectrical impedance (BIA): Body composition was assessed in the first 2 weeks of admission allowing a time-lapse for the stabilization of fluid and electrolyte status, likely to be affected by abnormal behaviors such as vomiting, purging, and diuretic abuse [27], [28]. The principles for measurement of body composition by BIA have been previously described by Kyle et al. [29]. BIA was measured using the Bioelectrical Analyzer (FORANA, Helios, Frankfurt, Germany) with an alternating electric current at 50 kHz and 800 mAmp and 4 skin electrodes (BIANOSTIC, DataInput, Darmstadt, Germany) positioned on the right wrist and ankle. The patient was lying in the supine position on a bed for the analysis and the skin was cleaned with 70% alcohol for better conductance. Resistance (R) and Reactance (Xc) in Ohms were determined.

Choice of BIA equation: We recently compared 5 BIA equations validated in normal populations with DXA (Dual- X-ray absorptiometry) in AN population [30]. We found that the Deurenberg equation [31] gave the better estimates of fat mass (FM) and fat free mass (FFM) compared to DXA, the reference method:
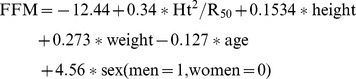



FM and FFM indices (FMI and FFMI): Usually, the percentage of body fat is used to adjust fat to bodyweight; However 2 individuals with different percentages of fat mass can have either identical FFM but different FM, or identical FM but different FFM [32]. Individual height variations in relation to FFM are not taken into account. In the general population the percentage of fat mass is an acceptable approximation but in AN, FM and FFM are not affected to the same extent due to the variable impact of factors such as physical activity, vomiting, laxative abuse and diet [14], [33]. Thus in the study by VanItally et al., [34] adjustment of FM and FFM on height was used to enable independent evaluation of both FM and FFM relative to stature: FFMI* = *FFM (kg)/ht (m^2^) and FMI* = *FM (kg)/ht (m^2^).

FFMI and FMI are relevant in studies comparing patients with controls, and also to determine new reference data on body composition [32]. In the present study, FFMI and FMI were used for FM and FFM because we believe that adjustment for height in a heterogeneous sample like ours is essential for unambiguous comparison.

Albumin and prealbumin: Blood samples were collected from all patients in each center on the day of admission to inpatient treatment. Albumin and prealbumin values were adjusted and expressed as ratio relative to the normal value on the basis of average standard values and testing methods for each centres.

Treatment: Information on current medication (at inclusion in the study) was collected from the medical teams in each centre for each patient. Antidepressants were selective serotonin reuptake inhibitors and anxiolytics were benzodiazepines and antihistamines.

### Statistical Analysis

Statistical analysis was performed on SPSS 17.0. First descriptive statistics were produced. Results are presented as means (SD). The relationships between the different psychological scores (BDI, HAD depression, HAD anxiety, MOCI and LSAS) and the nutritional status indicators (BMI, FFMI, FMI, severity of weight loss, albumin and prealbumin levels) were tested using Pearson’s correlations. A statistical significance was considered when p was <0.05. Potential confounding factors (minimum lifetime BMI, age and duration of illness) that might affect psychological symptoms and nutritional status, were also included in the analyses Student t-tests were performed in order to check for differences in nutritional status in relation to potential confounding factors such as restrictive and binge/purging AN, presence/absence of drug treatment (antidepressants, anxiolytics). Following this, multiple linear regression models were run in order to test the link between psychiatric scale scores and the nutritional status indicators, adjusting on all confounding factors identified by univariate analysis. Thus each of the psychological scores was a dependent variable, and the model had the following independent variables: age, medication (antidepressants and anxiolytics) for adjustment, and BMI, FFMI, FMI, severity of weight loss, albumin level and prealbumin level as nutritional indicators.

## Results

### Sample Characteristics

We recruited 155 subjects, 74 patients were restrictive-AN type (AN-R) (47.7%) and 81 were binging-purging-AN type (AN-BP) (52.3%). Concerning medication, 70 patients (45.2%) were not receiving any antidepressant or anxiolytic treatment, 57 patients (36.8%) were on antidepressants, 60 patients (38.7%) were on anxiolytics, and 32 patients (20.6%) were on both antidepressants and anxiolytics (percentage is above 100 as some of the patients are counted in more than one group).

The clinical characteristics of all 155 subjects at inclusion are presented in table 1.

**Table 1:** Patient (n = 155) characteristics at inclusion.

	Mean	SD	Minimum	Maximum
**Age (years)**	20.90	6.16	13.16	43.76
**Inclusion BMI (kg/m^2^)**	14.43	1.46	10.72	18.51
**Minimum lifetime BMI** **(kg/m^2^)**	13.05	1.55	8.59	18.51
**Maximum lifetime BMI** **(kg/m^2^)**	20.08	3.24	13.15	32.47
**Severity of weight loss**	5.82	3.22	0	17.94
**Duration of illness (years)**	4.29	4.71	0.12	24.39
**FFMI** +	12.54	0.8	10.68	15.03
**FMI** +	1.92	1.14	−0.53*	5.10
**Albumin level** #	1.02	0.16	0.70	1.53
**Prealbumin level** #	0.81	0.21	0.06	1.36

BMI: Body Mass Index; SD: Standard Deviation.

* Negative values are due to negative body fat predicted values because of the severe emaciation of AN patients.

+ FFMI and FMI are obtained for 146 patients.

# Albumin and prealbumin were measured for 132 patients.

Global scores for the psychological scales are presented in table 2. For example the BDI average score is 26.8 for our AN sample. In the BDI, 0–9 indicates minimal depression, 10–18 indicates mild depression, 19–29 indicates moderate depression and 30–63 indicates severe depression [20]. The LSAS average score was 57.7 for the fear/anxiety items alone (without summing responses), which puts these patients in the severe social phobia category [35].

**Table 2:** Psychlogical scores at inclusion.

	Mean	SD	Minimum	Maximum
**BDI**	26.80	11.73	3.00	53.00
**HAD depression**	9.43	4.52	.00	18.00
**HAD anxiety**	12.58	4.35	2.00	21.00
**MOCI**	12.53	5.80	1.00	28.00
**LSAS**	57.73	15.85	26.00	95.00

SD: standard deviation; BDI : Beck Depression Inventory, HAD: Hospital Anxiety and Depression scale, MOCI : Maudsley Obsessive-Compulsive Inventory, LSAS: Liebowitz social anxiety scale.

### Relationship Between Psychological Symptoms and Malnutrition Indicators

No correlation was found between the nutritional markers at inclusion (i.e. BMI, fat-free mass index, fat mass index, or severity of weight loss) with any of the psychological scores Albumin levels were negatively correlated to LSAS scores (p = 0.004; r = −0.247).

1. Potential confounding factors

Patients’ psychological scores and nutritional status indicators were correlated with AN subtype, type of treatment, age, and minimum lifetime BMI. Only significant results are reported, details can be requested from the authors.

AN-BP and AN-R had no significant difference in psychometric scores. Differences in body composition and BMI are shown in table 3.

**Table 3:** Differences and means of BMI and body composition components between AN-R and AN-BP.

	Mean±SD AN-R (N = 74)	Mean±SD AN-BP (N = 80)	p
**Inclusion BMI (kg/m^2^)**	14.01±1.16	14.8±1.6	0.001
**Minimum lifetime BMI (kg/m^2^)**	12.6±1.25	13.46±1.68	0.000
**Maximum lifetime BMI (kg/m^2^)**	19.49±3.34	20.63±3.05	0.03
**FMI** +	1.61±0.91	2.21±1.25	0.001
**FFMI** +	12.43±0.71	12.64±0.83	0.3

AN-R: Anorexia Nervosa Restrictive type.

AN-BP: Anorexia Nervosa Binge Purging type.

BMI: Body Mass Index; SD: Standard Deviation.

+ FFMI and FMI are obtained for 146 patients.

Patients on antidepressants were significantly older than those who were not (22.4±6.4 versus 20.0±5.9; p = 0.013), they had longer duration of illness (respectively 5.44±5.17 versus 3.53±4.27; p = 0.015) and they had significantly higher levels of depression measured on both the BDI (respectively 32.49±11.15 versus 23.42±10.75; p = 0.000) and the HADs (respectively 11.22±4.32 versus 8.36±4.31; p = 0.000); they had higher levels of anxiety (respectively 14.01±3.9 versus 11.72±4.40; p = 0.001) obsessive disorders (respectively 13.91±5.78 versus 11.70±5.68; p = 0.023) and social phobia (respectively 64.17±15.35 versus 53.96±14.96; p = 0.000). Lower albumin levels were detected for patients on antidepressant treatment than for those without antidepressants (respectively 0.98±0.16 versus 1.04±0.15; p = 0.035).

Compared to patients without anxiolytic treatment patients on anxiolytics had higher levels of depression measured by both the BDI (respectively 29.72±11.02 versus 24.95±11.83; p = 0.014) and the HADs (respectively 10.72±4.34 versus 8.62±4.46; p = 0.005).

Age was highly correlated to duration of illness in our sample (p = 0.000, r = 0.668) so we adjusted our comparison on age but not on duration of illness. Correlations between age, duration of illness and psychological scores, BMI and body composition are shown in table 4.

**Table 4:** Correlations between age, duration of illness and BMI, body composition components and psychological scores.

		Age	Inclusion BMI (kg/m^2^)	Durationof illness	FFMI	FMI	HAD anxiety	HAD depression	BDI	LSAS	MOCI
Age	r	1	−.002	.668*	−.239*	−.239*	.173*	.195*	.155*	.210*	−.008*
	p		.980	.000	.004	.004	.033	.0016	.055	.009	.919
Duration of illness	r	.668*	.002	1	−.238*	−.238*	.174*	.236*	.212*	.250*	.034*
	p	.000	.980		.004	.004	.032	.003	.009	.002	.680

BDI : Beck Depression Inventory, HAD: Hospital Anxiety and Depression scale, MOCI : Maudsley Obsessive-Compulsive Inventory, LSAS: Liebowitz social anxiety scale; BMI: Body Mass Index; SD: Standard Deviation;

+ FFMI and FMI are obtained for 146 patients.

Minimum lifetime BMI was not correlated to any of the psychological scores.

Finally only age and medication, but not subtype of AN nor minimum lifetime BMI, were introduced into the multivariate analysis as confounding factors because they were the only 2 factors that affected the psychological scores.

### Multivariate analysis

None of the psychological scores were explained by the nutritional indicators except for a negative correlation between albumin level and the LSAS fear scale (p = 0.024; beta = −0.225), as in the univariate analysis. Only antidepressants explained the variability in BDI scores (p = 0.029; beta = 0.228) and anxiolytics explained the variability in the HADs depression scores (p = 0.037; beta = 0.216).

## Discussion

The present study has adopted a very novel approach to investigating the relationship between nutritional status and psychological symptoms in AN, since it takes into account body composition and biological markers, and not solely weight or BMI, for the nutritional assessment, also adjusting for age and psychotropic treatment. To our knowledge it is the largest study on this topic in AN.

The most important finding of this study of a large sample of severe AN patients is that we did not identify any correlation between the level of depression, anxiety or Obsessive-compulsive disorder (OCD) and any measure of current nutritional status at inclusion, even when taking into account potential confounding factors (age and current psychotropic treatment). This is an unexpected result. Scores for the intensity of depression were however related to the presence of psychotropic treatment and in certain cases to age or duration of illness (univariate analysis).

Three main hypotheses might explain our results that failed to reveal any correlation between the nutritional status and the psychological symptoms that we measured.

First of all, although we studied a large sample, the subjects’ characteristics might have been largely homogeneous in terms of the severity of malnutrition and the reported psychological symptoms, so that we could not identify any relationship between them. Patients were evaluated at admission to inpatient treatment at the most (or one of the most) severe moments in their illness. For example the narrow SD for BMI (mean = 14.43; SD = 1.45) shows the severe malnutrition and the relative homogeneity in the sample. Even the patients that had higher BMI had lost huge amounts of weight (Cf. methods) and thus were all severely malnourished. Consequently there was very little variation in the degree of malnutrition. Despite the fact that levels of depression and anxiety were variable, we were not able to establish any link with levels of malnutrition. There may be a nutrition threshold, whereby psychological state is only affected when a certain degree of nutritional deficiency has been reached.

Second, we evaluated nutritional status in more comprehensive manner and in a larger sample compared to previous studies, and we used relatively a large set of indicators. However, body composition was measured using the BIA which is not a reference method (such as Dual-emission X-ray absorptiometry (DXA) or measurements using 4 compartment models). The severely malnourished status of the patients did not enable transfer to DXA centres for the measures to be performed. Also, the severity of malnutrition was measured by a rough estimation of the difference between the highest lifetime BMI and BMI at inclusion, thus considering weight loss to be linear, and not accounting for duration of illness and weight fluctuations. A more precise measure of these variations should be performed to provide information on this subject.

Third, as hypothesised by certain authors [36], [37] depression in AN, rather than having a single aetiology, is likely to be the consequence of various factors; depressive and anxiety symptoms in severely malnourished AN patients could therefore be mainly due to factors other than malnutrition, such as depressive symptoms linked to exhaustion, chronic illness or in some cases premorbid depression.

An interesting yet worrying observation from our study was the frequent use of psychotropic drugs in the treatment of very malnourished patients. More than 36% percent of AN patients admitted were receiving antidepressants. This is unusual, especially in severely malnourished subjects: it is well established that antidepressants are not effective on patients with low BMI [2]. These treatments have usually been prescribed before inpatient admission, generally by non-specialized physicians, and they are generally stopped after admission, because they are ineffective. Despite these elements, it is interesting to see that the higher the anxiety or depressive scores, the more likely patients are to be receiving anti-depressants (AD).

How can we understand the link between psychological symptoms and malnutrition in the light of our data and the literature?

In the first stages of the illness, patients report that starvation provides relief from pre-existing anxiety and depressive symptoms. However, in a second stage, these symptoms tend to increase and regardless if malnutrition and starvation continue. If at the beginning of the illness, patients feel better and less anxious although they are starving, this might be due to complex biological and psychological mechanisms: depletion in tryptophan resulting from a strict diet could relieve anxiety, as suggested by Kaye et al. [38]. In addition, other effects such as satisfaction at having lost weight, positive reinforcement from peers [39], or battling against hunger as a source of pleasure and control [40], enable them to experience a degree of “well-being”. However, with time, these effects fade and anxiety and depression re-emerge, along with other rituals and obsessions. It is at this stage that patients are usually admitted to hospitalization. This anxio-depressive recrudescence is also explained by several other factors: the regulation and adaptation of the body to all kinds of nutritional deficiencies and hormonal changes, negative comments concerning extreme thinness, exhaustion, chronicity of the illness and the hospitalization itself. Thus the patient can be caught in a vicious circle that drives him/her to ever-lower BMI, sometimes fatal. In fact, the patient adopts again the first strategy, which is starvation, in an attempt to decrease anxiety and depression, as at the beginning of the illness. Unfortunately, this strategy aggravates the anxiety and depression and the vicious circle described by Garner described [39] becomes established.

### Conclusions

The present study is a pioneer investigation of relationships between various nutritional indicators and psychological symptoms in severely malnourished AN patients. In contrast with theories set out in the literature, we did not identify any correlations between severely malnourished status and psychological symptoms. However these results suggest several lines of research to confirm this finding. The use of even better nutritional indicators is needed, for example DXA instead of BIA for body composition analysis, and other than albumin and prealbumin proteins as serum markers [41]. The development of a precise measure of the scale of weight loss could be beneficial. Screening for vitamin and minerals levels could also help to distinguish symptoms resembling depression or anxiety, such as irritability, moodiness, restlessness, etc, associated with malnutrition (vitamin deficiencies, mineral depletion and decreased food intake [42]–[45]). These could mediate the effect of malnutrition on psychological symptoms more markedly than the variables explored in this study. Clinicians and the treating team of AN, should be aware that there could be confusion in the aetiology of certain malnutrition symptoms that appear as depression and anxiety symptoms.

The cornerstone of treating AN is still nutrition rehabilitation which should be initiated immediately [2]. Nutrition rehabilitation should start first in order to decrease immediately physical complications and psychological well-being. In practice managing co-occurent anxiety or depression symptoms in ED patients will include the specific treatment of ED, that could lower a part of anxiety and depressive symptoms by nutrition rehabilitation, withdrawal from binges and purges, specific psychotherapy (individual or family therapy) and work on the social impact of the illness.

Future studies with a longitudinal design and a follow up on the evolution during treatment are needed to explore variations in nutritional status in relation to psychological symptoms using more heterogeneous samples. For instance, future research should consider including a group of healthy controls or a group of recovered subjects, and the use of further assessment scales for psychological symptoms. More studies are needed to confirm our results.
