# Correction: Relationship between Affective Symptoms and Malnutrition Severity in Severe Anorexia Nervosa

**DOI:** 10.1371/annotation/2590ee07-f8c4-42c7-8ccd-f3b8ca79c59c

**Published:** 2013-10-04

**Authors:** Lama Mattar, Caroline Huas, EVHAN group, Nathalie Godart

There was an error in the affiliation for author Caroline Huas. The affiliation should appear as: INSERM U669, Maison de Solenn, Paris, France, Université François Rabelais de Tours, Département universitaire de médecine générale de médecine générale, Tours, France. 

